# Complex portal 2025: predicted human complexes and enhanced visualisation tools for the comparison of orthologous and paralogous complexes

**DOI:** 10.1093/nar/gkae1085

**Published:** 2024-11-18

**Authors:** Sucharitha Balu, Susie Huget, Juan Jose Medina Reyes, Eliot Ragueneau, Kalpana Panneerselvam, Samantha N Fischer, Erin R Claussen, Savvas Kourtis, Colin W Combe, Birgit H M Meldal, Livia Perfetto, Juri Rappsilber, Georg Kustatscher, Kevin Drew, Sandra Orchard, Henning Hermjakob

**Affiliations:** European Molecular Biology Laboratory, European Bioinformatics Institute (EMBL-EBI), Wellcome Genome Campus, Hinxton, Cambridge CB10 1SD, UK; European Molecular Biology Laboratory, European Bioinformatics Institute (EMBL-EBI), Wellcome Genome Campus, Hinxton, Cambridge CB10 1SD, UK; European Molecular Biology Laboratory, European Bioinformatics Institute (EMBL-EBI), Wellcome Genome Campus, Hinxton, Cambridge CB10 1SD, UK; European Molecular Biology Laboratory, European Bioinformatics Institute (EMBL-EBI), Wellcome Genome Campus, Hinxton, Cambridge CB10 1SD, UK; European Molecular Biology Laboratory, European Bioinformatics Institute (EMBL-EBI), Wellcome Genome Campus, Hinxton, Cambridge CB10 1SD, UK; Department of Biological Sciences, University of Illinois at Chicago, Chicago, IL 60607, USA; Department of Biological Sciences, University of Illinois at Chicago, Chicago, IL 60607, USA; Centre for Genomic Regulation, Barcelona, Spain; Wellcome Centre for Cell Biology, University of Edinburgh, Edinburgh EH9 3BF, UK; Pfizer UK, Dorking Rd, Tadworth KT20 7NY, UK; University of Rome La Sapienza, department of Biology and Biotechnologies “C. Darwin”, Rome, Italy; Department of Biological Sciences, University of Illinois at Chicago, Chicago, IL 60607, USA; Technische Universität Berlin, Chair of Bioanalytics, 10623 Berlin, Germany; Wellcome Centre for Cell Biology, University of Edinburgh, Edinburgh EH9 3BF, UK; Department of Biological Sciences, University of Illinois at Chicago, Chicago, IL 60607, USA; European Molecular Biology Laboratory, European Bioinformatics Institute (EMBL-EBI), Wellcome Genome Campus, Hinxton, Cambridge CB10 1SD, UK; European Molecular Biology Laboratory, European Bioinformatics Institute (EMBL-EBI), Wellcome Genome Campus, Hinxton, Cambridge CB10 1SD, UK

## Abstract

The Complex Portal (www.ebi.ac.uk/complexportal) is a manually curated reference database for molecular complexes. It is a unifying web resource linking aggregated data on composition, topology and the function of macromolecular complexes from 28 species. In addition to significantly extending the number of manually curated complexes, we have massively extended the coverage of the human complexome through the incorporation of high confidence assemblies predicted by machine-learning algorithms trained on large-scale experimental data. The current content of the portal comprising 2150 human complexes has been augmented by 14 964 machine-learning (ML) predicted complexes from hu.MAP3.0. We have refactored the website to enable easy search and filtering of these different classes of protein complexes and have implemented the Complex Navigator, a visualisation tool to facilitate comparison of related complexes in the context of orthology or paralogy. We have embedded the Rhea reaction visualisation tool into the website to enable users to view the catalytic activity of enzyme complexes.

## Graphical Abstract

**Graphical Abstract:**
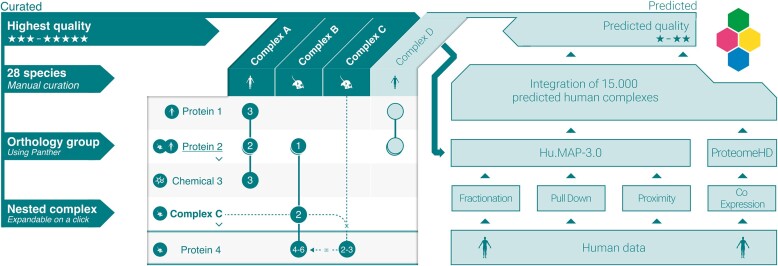

## Introduction

Proteins drive biological processes through both stable and transient interactions with other proteins. Proteins which bind together to form stable macro-molecular assemblies, or complexes, act as a single entity to perform their biological function. The Complex Portal (www.ebi.ac.uk/complexportal) (1) serves as a unifying resource, linking information on protein complexes across multiple resources by providing stable identifiers and summaries of the molecular composition, topology (when known) and function of stable macromolecular complexes from a selection of model organisms and organisms of special interest. Complexes described within the Complex Portal are defined as containing two or more proteins but can also contain other molecules such as nucleic acids or ligands which contribute to the function and stability of the complex. Participants within the complex have been maintained using stable identifiers with UniProt (2) IDs referencing proteins, RNAcentral (3) IDs for nucleic acids and the chemical ontology ChEBI (4) to represent small molecules. We have continued to add to and enrich the dataset with cross-references linking related information. For example, there are currently 211 complexes cross-referenced to ChEMBL (5) drug targets, 1279 to PDB (6) and 275 to EMDB (7) structural records, as well as 825 to the Reactome (8) pathway database (Table 1). Rhea (9) is a curated resource describing, from literature, enzyme-catalysed and spontaneously occurring biological reactions which utilises ChEBI references. Rhea cross-references, new at the time of our previous publication, have continued to be added to catalytically active complexes to describe reaction participants, their chemical structures and chemical transformations, thus providing a more granular description of the enzyme/substrate interaction. In September 2023, the Complex Portal embedded a visualisation widget developed by the Swiss Institute of Bioinformatics (SIB) to visualise reactions from Rhea. This has allowed us to display Rhea reactions cross-referenced to complexes as part of the Catalytic Activity section of an entry.

**Table 1.** Complex Portal cross-references to databases with related information across all species

Database	Number of complexes
IMEx experimental evidence^a^	1755
wwpdb	1279
EMDB	275
ChEMBL target	211
Enzyme Commission numbers	2185
Reactome	825
Rhea	1058

^a^Confirmatory experimental evidence curated by members of the IMEx Consortium (10) or confirmed by structural studies.

The Complex Portal has steadily increased its coverage of complexes of model organisms and as of build 247 (20 May 2024), complexes from 28 species have been curated and released. A summary of the increase in coverage of selected organisms is described in Table 2. The Complex Portal is supported by contributions from several model organism-specific databases including complexes described by FlyBase (11) for *Drosophila melanogaster*, PomBase (12) for *Schizosaccharomyces pombe* and Saccharomyces Genome Database (SGD) for *Saccharomyces cerevisiae* (13). We continue to maintain and update any new evidence to our recently completed *S. cerevisiae* and *Escherichia coli* complexomes as it becomes available.

**Table 2.** Increased coverage of curated complexes in selected species

Organism	Build 241 (13 October 2021)	Build 247 (20 May 2024)
*Homo sapiens*	1255	2152
*Drosophila melanogaster*	21	243
*Escherichia coli* (K12)	322^a^	324
*Saccharomyces cerevisiae*	616^a^	628

^a^previously completed first draft of *S. cerevisiae* and *E. coli* complexomes, for details see PMID: 33677561 (14) and PMID: 34718729 (15).

## Content

### Curation update

Since our previous publication (15), the focus of our curation efforts has been directed towards mapping the much sought after human complexome; our coverage of which has almost doubled during this period to >2150 manually curated complexes. We have continued to maintain a broad, unbiased coverage of the human complexome and have paid particular attention to families of complexes whose members, in addition to their shared function, for example a common enzymatic activity, are involved in distinct biological processes, such as members of the proteasome family. With that in mind, our manual curation efforts include complexes which contain participants that differ at the isoform level or variant forms with a probable common ancestor as well as tissue-specific complexes. Some examples of the level of granularity we capture include:

- *Isoforms:* Capturing complexes at the isoform (or pro-chain) level has allowed us insight into the functionally contrasting roles played: an example case is the IL6 family of cytokine receptor-ligand assemblies (CPX-623, CPX-8936, CPX-8967, CPX-8968, CPX-8969), where, dependent on the protein components of the receptor-ligand complex, IL6 mediates either a pro-inflammatory trans-signalling pathway (CPX-8967) or a classical anti-inflammatory signalling cascade (CPX-623).
- *Complex Variants:* A comparison of different forms of the proteasome regulated by two variant members of the PA28 family of regulatory lids PSME1-PSME2 and PSME3 described in CPX-9002 and CPX-9001, respectively captures the specialist roles the proteasomes play in MHC Class 1 peptide presentation versus cell growth, proliferation and apoptosis.
- *Tissue-specific complexes:* The Complex Portal also captures tissue-specific complex variants where possible. An example of this is the 20S Thymoproteasome (CPX-9004). The subunit-specific tissue expression of PSMB11 in the 20S Thymoproteasome in place of PSMB6 in the 20S Proteasome (CPX-8806) results in them being involved in different biological processes with the 20S Thymoproteasome having a role in CD8 + repertoire formation.

### Content expansion

**Figure 1.**
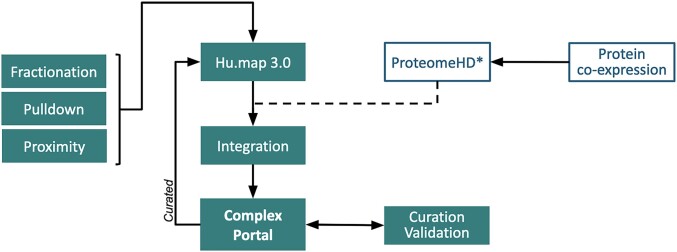
Integration of ML-predicted complexes into the Complex Portal. *ProteomeHD (18) data will be included in a future release of the database (see future plans).

Whilst we have continued to significantly expand our coverage of the human complexome through high quality manual curation, in an effort to rapidly increase our coverage and more fully map the potential array of cellular assemblies, a collaboration was established with Kevin Drew, University of Illinois Chicago, and Georg Kustatscher, University of Edinburgh (Figure 1). hu.MAP2.0 (16) was previously the most comprehensive map of computationally inferred, high confidence human complexes (http://humap2.proteincomplexes.org/about) derived by machine-learning algorithms. hu.MAP2.0 has now been extended by additional large-scale mass spectrometry affinity purification, co-fractionation and proximity ligation experiments, and a training/test set of 2150 high quality manually curated Complex Portal human complexes. The resulting 15 350 predicted complexes were clustered by confidence scores to create hu.MAP3.0 (17).

The resulting hu.MAP3.0 clusters were imported into the Complex Portal and matched at the canonical level to existing manually curated entries. No thresholds were imposed in the minimum number of participants required to make the match. This resulted in:

- 74 exact matches where hu.MAP3.0 clusters have a 100% match at the protein level to existing manually curated entries. These were merged and are displayed with a hu.MAP3.0 cross-reference added;
- 214 hu.MAP3.0 clusters partially mapped to existing Complex Portal complexes. Partial matches reflect cases where all the proteins suggested by ML are present in the Complex Portal entry but additional proteins are present in the manually curated version. All these were re-checked by a biocurator and it was decided that 206 will be added as a subset to the relevant manually curated entry with hu.MAP3.0 cluster ID and a qualifier directing the user to the parent complex with immediate effect; a further 9 are still under investigation. Partial matches where hu.MAP3.0 clusters contain additional novel protein components were treated as novel Complex Portal entries.
- 14 964 new ML-predicted complexes. As on-going practice in all future hu.MAP iterations, hu.MAP cluster IDs will be added to the Complex Portal entries and matched at the canonical sequence level as soon as new data becomes available. Novel complexes and cases where ML suggests additional components to existing manually curated complexes will be validated by manual curation, wherever possible, and entries will be updated accordingly in future releases.

### Data stability

We maintain stable complex and participant identifiers for our curated complexes, and we will extend this practice such that in the event of complexes being deleted from a hu.MAP version, we will continue to maintain them. Deleted complexes will be excluded from browse functionality and marked as ‘not supported by current external data sources anymore’ but will remain accessible from the Complex Portal through their accession number. Thus, all Complex Portal accession numbers will remain referenceable, ensuring that the Complex Portal can preserve referential integrity and fulfil its role as a reference resource for molecular complexes in other databases, similar to the role e.g. UniProt implements for protein sequences.

### Evidence for the predicted complexes

Evidence and Conclusion Ontology (ECO) codes (19) are key to describing the weight of evidence supporting Complex Portal entries (summarised in Table 3; described in full on the documentation tab of the website). We have extended the current tiered system with additional terms to describe complexes only predicted to exist by machine-learning algorithms and those with additional computational evidence-based data on, for example, co-variation (Table 3). For ease of usability, the ECO codes are illustrated by a 1–5 star-rating system.

**Table 3.** Evidence and Conclusion Ontology (ECO) codes used to indicate the type of evidence for the existence of a complex

ECO code	Describes evidence derived from	Confidence score
ECO:0000353	Physical interaction evidence	5
ECO:0005543	Experimental evidence from mixed species	5
ECO:0005610	Inferred based on homology	4
ECO:0005544	Inferred based on orthology	4
ECO:0005546	Inferred based on paralogy	4
ECO:0005547	Inferred by curator	3
ECO:0007653	ML-predicted complex with additional/combinatorial evidence	2
ECO:0008004	ML- predicted complex	1

**Figure 2.**
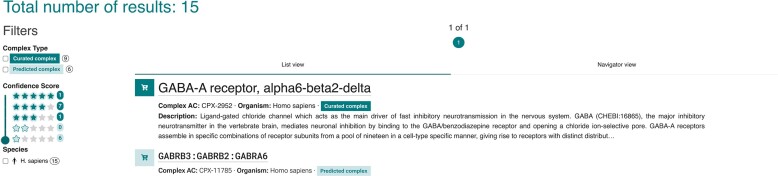
Complex Portal search results with filter options displayed.

A filter has been added to allow the search, selection or removal of the predicted complexes from selected datasets enabling the user to select complexes by ‘Complex Type’ or by ‘Confidence Score’ which describes the assigned ECO code(s) (an example search is shown in Figure 2).

Future plans include filtering of predicted complexes on confidence values assigned by hu.MAP3.0 or by another data source. In time, the entire prediction pipeline described in Figure 1 will be developed as a highly automated, adaptable and repeatable workflow which will be continuously updated and expanded to allow the set of inferred complexes to evolve as additional data becomes available for human and additional species.

Work has begun to survey the hu.MAP3.0 clusters for: (i) predicted complexes not in the Complex Portal but could be annotated with experimental evidence from IntAct or from the literature, as well as for (ii) overlaps between manually-curated and predicted complexes to substantiate evidence of potential sub-complexes.

## Visualization

The Complex Portal provides an adaptive visualisation interface; depending on the number of search results, one of three possible visualisations is provided.

### Complex viewer

**Figure 3.**
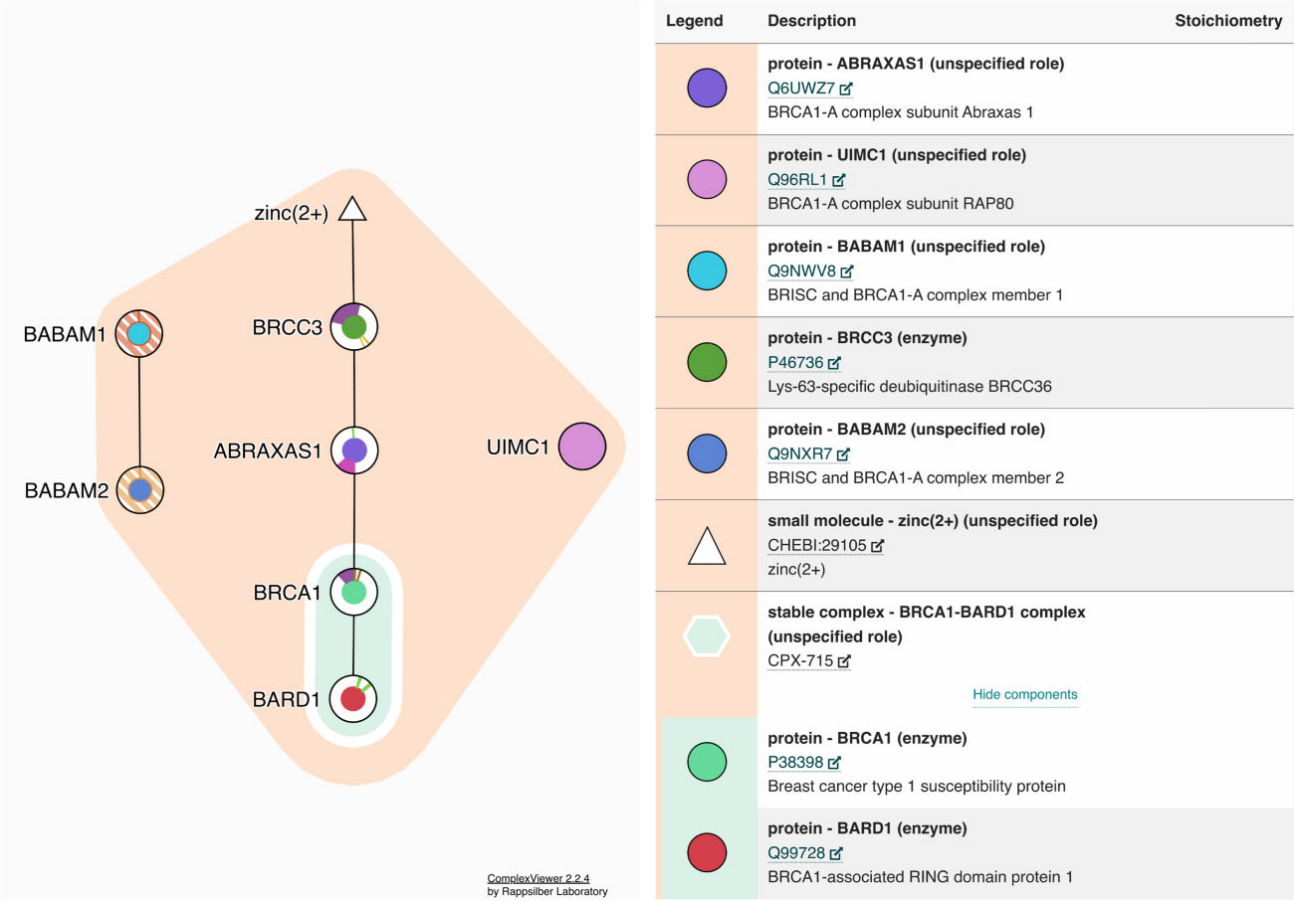
The left-hand side panel and right-hand side panel describe the topological view and legend, respectively, of the BRCA1–BARD1 sub-complex (CPX-715) nested within the BRCA1-A complex (CPX-4425). The nested sub-complex within the complex is depicted by the extended orange vertical line in the legend.

If the current search results in a single complex, or if the user has clicked on a specific complex accession number, the ‘Complex Viewer’ (20) is used to provide a feature-rich view of the individual complex. The Complex Viewer is a visualisation tool that displays complexes of varying size while preserving their internal topology, binding features and stoichiometry. The participant table in the Complex Viewer has been updated to display hierarchically nested complexes and the participants within subcomplexes. Users can now expand subcomplexes to view its participants; and these are colour-coordinated within the Complex Viewer (Figure 3). This feature is useful in visualising the relationship between the unique roles played by subcomplexes and larger holo-complexes.

### Complex navigator

**Figure 4.**
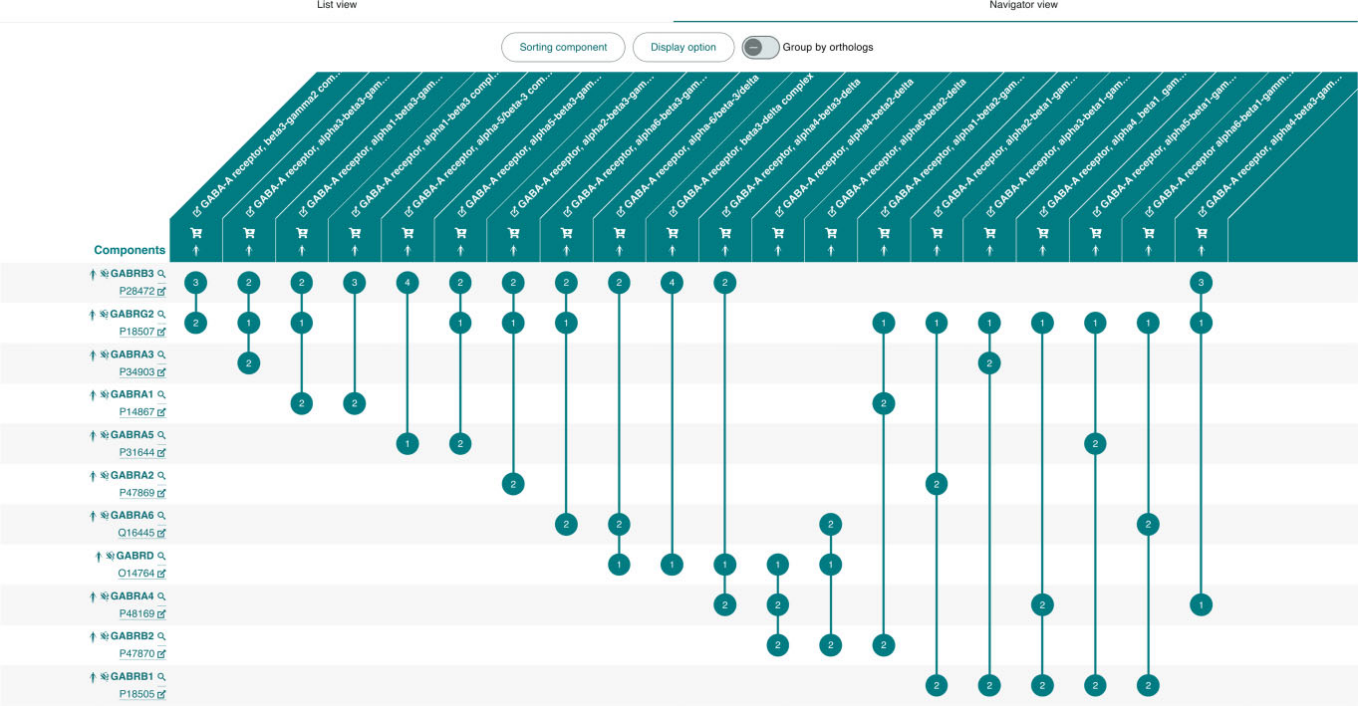
The Complex Navigator View comparing selected GABA-A complexes. Further information on participants can be accessed by clicking the magnifying glass icon. This generates a query to the participant's respective UniProt, ChEBI, RNAcentral or Complex Portal pages. Participants are sorted by occurrence by default, but they can also be sorted by type, by organism, or by gene orthology. If a non-default sorting option is selected, a sidebar appears on the left of the table, indicating the organism or type and enhancing the connection between them. The Complex Navigator is set by default to ‘detailed’ display, but users can choose their preferred display mode. The ‘compact’ display allows the comparison of a greater number of participants by hiding some participant attributes. In this mode, the participant's name becomes clickable, generating a new query for it. Complexes can be participants of other complexes, in this case clicking on them in the participant column will extend the view to show all participants of the respective complex. For six to twenty complexes the names are shown at a 45-degree angle to provide a more compact display.

For result sets of ‘a few’ complexes, by default from two to twenty, we have developed a new visualisation component, the Complex Navigator, enabling comparison of multiple related complexes and their participants (Figure 4). The representation within the Navigator is inspired by UpSet plots (21) and displays a matrix where rows represent complexes, columns their respective interactors, and cells contain the interactor's stoichiometry within a complex (if known). The Navigator displays manually curated complexes first, followed by predicted complexes. The Complex Navigator allows comparison of complexes containing paralogous proteins as well as orthologous complexes, making it useful in determining common/unique functionality between paralogous or orthologous complexes which may inform their evolution from a common ancestor.

### List view

For queries resulting in >20 complexes, the classic List View (Figure 2) is used. It presents a paginated view for larger result sets, with a brief description of each complex.

Users can switch between the Complex Navigator and List View through the options in the page header. If the Complex Navigator is explicitly selected for >20 complexes, it will also use a paginated view.

Search results in both the Complex Navigator and List View can be further filtered by newly added options indicated by icons denoting ‘Species’, ‘Biological Role’ and ‘Component Type’. Complexes can also be further filtered to include ‘Curated’ and/or ‘Predicted’, as described above. Complexes can be selected using a ‘trolley’ icon displayed next to the complex's name which collates them in the ‘basket’ accessible in a tab at the top of the page. The basked can be clicked, generating a new view, for example, to make direct comparisons between selected complexes.

## Infrastructure improvements

UniProt, our main reference database for proteins, refactored its website and APIs during 2022 affecting many of our curation processes and release pipeline. Our dependency on UniProt centres on the usage of a java API (uniprot-japi). An overall technology stack update and unification of legacy code base on the Java 11 (required to read the latest uniprot-japi, version 1.1.2) was implemented and deployed. The migration of our code base to Java 11 was completed in February 2023 and we currently use the uniprot-japi version 1.1.2.

## Summary and future plans

Our key advances since the last Complex Portal publication are the doubling of curated human complexes, the inclusion of around 15, 000 predicted hu.MAP3.0 complexes, and the introduction of the Complex Navigator visualisation component. The successful aggregation of curated and ML-predicted complex data has paved the way for us to include complex data from other experimental, curated, or ML-derived sources in the future.

The Complex Portal is already referenced by multiple databases including UniProtKB, members of the IMEx Consortium, the Gene Ontology (22,27) and several model organism data resources and is integrated into the Open Targets (23) partnership together with molecular interaction data from IntAct (24), Reactome (9) and SIGNOR (25). The increase in content of human complexes now available in the Complex Portal will thus be available for systematic drug target identification. Our future plans include:

- *ProteomeHD:* Protein covariation (co-expression) data from ProteomeHD (18) and ProteomeHD.2 (Kourtis *et al.*, in preparation) will be used to augment the hu.MAP3.0 predicted complexes. Protein covariation will serve as orthogonal experimental evidence to support hu.MAP3.0 confidence scores, and to distinguish between core complexes and those expressed in a context-specific manner, providing an additional rich layer of data.
- *PRIDE crosslinking archive* (https://www.ebi.ac.uk/pride/archive/crosslinking): data from crosslinking mass spectrometry (MS) experiments will be ported as streamlined biochemical interaction evidence for binary protein–protein interactions from the PRIDE crosslinking archive.
- *PSI-COM:* we are working with the molecular interaction workgroup of the HUPO-PSI to ensure that data formats and standards enable the comparison of complexes under differing cellular environments to capture context-specific data on biological function and process.
- *Network analysis with Cytoscape:* the increased coverage of human complexes will be exploited for network analysis of complexes in the Cytoscape application ClueGo (26) using GO (22,27) enrichment tags.
- *AlphaFold Multimer:* where possible, AlphaFold Multimer (28) assemblies will be integrated and displayed as a widget on the details page of a complex.
- *Other model organisms:*We plan to extend the pipeline to generate predicted complexes for other model organisms, for example, *Arabidopsis thaliana*.

## Data Availability

The Complex Portal is a community project. Developers can contribute to the code at https://github.com/Complex-Portal/complex-portal-view (archived at https://doi.org/10.6084/m9.figshare.27284529). Data can be accessed either via our ftp site (ftp.ebi.ac.uk/pub/databases/intact/complex/current/) or our REST API (https://www.ebi.ac.uk/intact/complex-ws/). The FTP site contains files in different formats, grouped by species, and for each species there are separate files containing manually curated complexes and ML-predicted complexes. The Complex Portal is an open source (Apache 2.0), open data (CC0) project, details on https://www.ebi.ac.uk/complexportal/about#license_privacy.
